# Cross-sectional study of social behaviors in preschool children and exposure to flame retardants

**DOI:** 10.1186/s12940-017-0224-6

**Published:** 2017-03-09

**Authors:** Shannon T. Lipscomb, Megan M. McClelland, Megan MacDonald, Andres Cardenas, Kim A. Anderson, Molly L. Kile

**Affiliations:** 1 Oregon State University Cascades, Bend, OR USA; 2 College of Public Health and Human Sciences, Oregon State University, 15 Milam Hall, Corvallis, OR 97331 USA; 3 Harvard T.H. Chan School of Public Health, Boston, MA USA; 4 Oregon State University, College of Agricultural Sciences, Corvallis, OR USA

**Keywords:** Triphenyl phosphate, Flame retardants, Polybrominated diphenyl ethers, Organophosphate, Tris, Children’s health, Externalizing behavior, Responsibility, Assertiveness

## Abstract

**Background:**

Children are exposed to flame retardants from the built environment. Brominated diphenyl ethers (BDE) and organophosphate-based flame retardants (OPFRs) are associated with poorer neurocognitive functioning in children. Less is known, however, about the association between these classes of compounds and children’s emotional and social behaviors. The objective of this study was to determine if flame retardant exposure was associated with measurable differences in social behaviors among children ages 3–5 years.

**Methods:**

We examined teacher-rated social behaviors measured using the Social Skills Improvement Rating Scale (SSIS) and personal exposure to flame retardants in children aged 3–5 years who attended preschool (*n* = 72). Silicone passive samplers worn for 7 days were used to assess personal exposure to 41 compounds using gas chromatography-mass spectrophotometer. These concentrations were then summed into total BDE and total OPFR exposure prior to natural log transformation. Separate generalized additive models were used to evaluate the relationship between seven subscales of the SSIS and lnΣBDE or lnΣOPFR adjusting for other age, sex, adverse social experiences, and family context.

**Results:**

All children were exposed to a mixture of flame retardant compounds. We observed a dose dependent relationship between lnΣOPFR and two subscales where children with higher exposures were rated by their preschool teachers as having less responsible behavior (*p* = 0.07) and more externalizing behavior problems (*p* = 0.03). Additionally, children with higher lnΣBDE exposure were rated by teachers as less assertive (*p* = 0.007).

**Conclusions:**

We observed a cross-sectional association between children’s exposure to flame retardant compounds and teacher-rated social behaviors among preschool-aged children. Children with higher flame retardant exposures exhibited poorer social skills in three domains that play an important role in a child’s ability to succeed academically and socially.

## Background

Early childhood is a key developmental period for learning appropriate social behaviors. Individual differences in externalizing behaviors, such as hyperactivity, inattention, aggressive, and oppositional behaviors, that emerge during early childhood often persist throughout childhood [1, 2]. Furthermore, children who exhibit more externalizing behaviors tend to struggle more in both academic and social domains [3–5] and are more likely to develop mental illness by adulthood [6]. In contrast, young children who show more positive social behaviors, such as cooperation, assertiveness, and self-control, tend to have more success in school [7] and show more positive school adjustment, motivation, and involvement in learning [8].

Much of the prior research on the etiology of social behavior in early childhood has focused on children’s social experiences, at home and preschool, as well as children’s genetics [9, 10]. Yet there is concern that chemicals commonly found in children’s environments may adversely influence social and emotional behavioral development [11]. For instance, epidemiological studies have reported that children with higher lead exposure have a greater probability of demonstrating negative social behaviors in toddlers, children, and young teens [12–14]. Exposure to bisphenol A (BPA), an endocrine disrupting chemical, has also been linked to behavioral outcomes in young children [15, 16]. Another study reported an association between polychlorinated biphenyl (PCB) exposure during development and a greater risk of exhibiting attention-deficit and autistic-like behavior in childhood [17].

There is also considerable interest in whether exposure to flame retardants can influence children’s behavioral development. Flame retardants, namely brominated flame retardants (BFRs) and organophosphate-based flame retardants (OPFRs) are widely used in furniture, building materials, plastics, and electronics to reduce their flammability in order to meet fire safety standards [18]. Biomonitoring studies show that BFRs have increased in people over time [19] and are almost an order of magnitude higher in the U.S. compared to European and Asian populations [20]. Children also appear to have greater exposure to flame retardants as reflected by having much higher levels of BFRs in their blood compared to their mothers [21]. Several prospective epidemiological studies report that in utero exposure or early life exposure to selected BDE congeners (e.g. BDE-28, -47, -99, -100, or -153) are associated with adverse neurological developmental, attention deficits, poorer behavioral regulation, or social competence in children [22–31]. Very little is currently known about how exposure to OPFRs affect children’s neurodevelopment or social behaviors. Although data from experimental studies indicate that tris(2-chloroethyl)phosphate (TCEP) and tris(1,3-dichloropropyl)phosphate (TDCPP) affect neurodevelopmental responses in experimental systems [32, 33].

The current study expands prior research on the association between flame retardants and social behaviors by building on an existing study of preschool aged children [34, 35]. The objective of the study was to examine the association between two different classes of flame retardants (PBDEs and OPFRs) and preschool children’s social behaviors as assessed by the Social Skills Improvement System - Rating Scales (SSIS-RS) which is a clinically relevant assessment [36]. The scores from SSIS-RS also capture normal variation in children’s behaviors that predicts success in academic and social domains [37]. Furthermore, this study controlled for important psychosocial stressors that negatively affect behavior [38].

## Methods

### Study population and procedures

From October 2012 to January 2013, ninety-two children between the ages of 3–5 years old were recruited from 28 preschool classrooms in two geographic areas of Oregon, USA. Written informed consent was obtained from the parent and/or legal guardian for all participants and from all preschool teachers prior to engaging in any study activity. Child assent was indicated by their engagement with the materials and/or project staff.

Researchers visited participants’ homes where parents completed a series of structured questionnaires to capture socio-demographic information (e.g. household income, parental education levels, race, etc.) and the home learning environment. During this visit, each child was given a silicone passive wristband sampler to wear around his/her wrist or ankle. This sampler was used to assess the child’s exposure to flame retardants. Parents were requested to have their child wear the wristband continuously for 7 days, although it could be taken off at night and placed next to the child’s bed on a table if preferred. After 7 days, parents were instructed to seal the wristband in the polytetrafluoroethylene (PTFE) bag, note the number of days the child actually wore the wristband on the chain of custody tag, and place it in the mail. The concentration of flame retardants measured in the silicone passive wristband sampler were also reported back to the participants. Children’s social behaviors were assessed using the Social Skills Improvement System - Rating Scales by their teacher in the preschools they were attending.

Of the 92 wristbands distributed to the children, 77 were returned for analysis. Of those, five samples were excluded due to parent report of substantial deviance from the protocol (e.g. never worn by the child, lost at school for several weeks, or went through the laundry). Additionally, three parents chose not to answer questions on the socio-demographic questionnaire, which left a final sample size of 69 children with complete data that were included in the final analyses.

### Ethical statement

All research activities were approved by Oregon State University’s Institutional Review Board. All parents gave informed written consent and children gave assent before partaking in any research activity. Results from the chemical results from the wristbands were returned to the parents. Due to the novelty of the exposure assessment methodology there were no other populations that could serve as a comparison for this study. Subsequently, parents were told which flame retardants were detected in their children’s sample and where their child’s exposure ranked within this group of children (e.g. in the lowest 25^th^ percentile, in the 25^th^ to 75^th^ percentile, or highest 25^th^ percentile). Parents were also given resources created by the Agency for Toxic Substances and Disease Registry where they could learn more about flame retardants and resources created by the Oregon Environmental Council’s Eco-Health Homes Checkup Kit where they could learn about how to reduce exposure to pollutants in their home including flame retardants.

### Social behavior assessment

The teacher form of the Social Skills Improvement System Rating Scale (SSIS-RS) was used to measure children’s social behavior in preschool classrooms. The SSIS-RS is a standardized assessment of social skills and problem behaviors for children ages 3–18 years, has strong psychometric properties, and measures both normative and clinically-relevant variation [36]. Each item asks teachers to indicate the frequency of children’s behaviors from 0 (never) to 3 (always). We examined seven subscales representing positive behaviors: Communication, Cooperation, Assertion, Responsibility, Empathy, Engagement, and Self-Control; and four subscales representing behavior problem domains: Externalizing, Bullying, Hyperactivity/Inattention, and Internalizing. Sample items of positive behaviors include “*follows classroom rules*” and “*expresses feelings when wronged*” and “*makes a compromise during a conflict.*” Sample behavior problem items include “*disobeys rules or requests*”, “*Is aggressive toward people or objects*”, and “*acts without thinking*”.

Our preliminary analyses indicated similar associations between flame retardants and the three subscales of externalizing behavior problems (externalizing, hyperactivity/inattention, and bullying). Subsequently, we utilized an aggregate of these three subscales in the final analyses. Preliminary analyses suggested, however, that the subscales of positive behaviors were associated differently to PBDEs and OPFRs. Thus, each of the individual subscales of positive behaviors were examined in the final analyses. Internal consistencies for the subscales ranged from .81 for assertion to .93 for self-control and the externalizing aggregate.

### Flame retardant exposure

The methodology and results for the detection of flame retardants in silicone passive sampling devices utilized in this study have been published elsewhere [34]. Briefly, silicone wristbands were purchased from a commercial retailer (24hourwristbands.com) and prepared following the method described in O’Connell et al. [39]. The solvent-cleaned wristbands were then packaged in clean, air-tight PTFE bags along with a chain of custody tag and given to parents along with instructions to have the child wear the wristband at all time for 7 days either as a bracelet or anklet. Parents were instructed to place the wristband on a table near the child’s bed if he/she did not want to wear the wristband while sleeping. Parents were then requested to re-seal the wristband in its PTFE bag, fill in the chain of custody tag with the number of days the wristband was worn by the child, and mail it back to Oregon State University using the provided pre-paid business envelope. The wristbands were then extracted and analyzed for 41 different flame retardant compounds using gas chromatography mass spectrophotometry.

This analysis focused on 11 compounds (PBDE-47, PBDE-99, PBDE-153, PBDE-154, PBDE-49, PBDE28 + 33, tris(1,3-dichloro-2-propyl) phosphate], TPP [e.g. triphenylphosphate], TCPP [e.g tris(1-chloro-2-propyl) phosphate], and TCEP [e.g. tris(2-chloroethyl) phosphate) which were detected in 60% or more of the wristbands. For compounds that were measured below the limits of detection, a value was assigned which was equivalent to the LOD divided by the square root of 2. The concentration of the chemical detected in the wristband was then divided by the number of days the wristband was reported to be worn which resulted in a unit of nanograms per gram silicone per day (ng/g-day). All the calibration standards were within 15% of the true value for all compounds on 12 separate days indicating good instrument performance. Information describing the limit of detection and quality control measurements for these samples has been described in detail elsewhere [34].

Congeners in the same class (e.g. 4 BFRs and 7 OPFRs) were highly correlated with each other (ρ_spearman_ > 0.40). Thus, we created a sum score for the different flame retardant classes and used this as our exposure index. Subsequently, ƩPBDEs is the total amount of PBDE-47, PBDE-99, PBDE-153, PBDE-154, PBDE-49, and PBDE28 + 33; whereas ƩOPFRs is the total amount of TDCPP [e.g. tris(1,3-dichloro-2-propyl) phosphate], TPP [e.g. triphenylphosphate], TCPP [e.g tris(1-chloro-2-propyl) phosphate], and TCEP [e.g. tris(2-chloroethyl) phosphate].

### Covariates

The primary caregiver of the child was asked to complete several structured questionnaires to collect information about socio-demographics and aspects of the home environment. Six variables were aggregated into a covariate to represent the family context: maternal education, paternal education, maternal employment, paternal employment, household income, and home learning environment. Parents reported their total years of education (e.g. 12 = completed high school; 16 = 4-yr college degree, etc.). Parents who reported that they were employed either part- or full-time were coded as “1”; others were coded as “0” for not employed. Annual household income was reported on a scale from 1 = less than 22,000 to 8 = 70,001 or more. The home learning environment was measured with 14 items from the Parenting Questionnaire [40] related to literacy and numeracy activities in the household (e.g. *how often do you read to your child*? *How often do you encourage your child to do math-related activities?*). Items were standardized and aggregated into a home learning composite (Cronbach’s alpha = 0.83). Values for all six variables were standardized and averaged (Cronbach’s alpha = 0.74). Previous research has documented similar internal reliabilities for the Parenting Questionnaire [40, 41].

Parents were also asked to self-report if their child had ever experienced any of the four following adverse experiences since birth: lived with an adult that had problems with alcohol or drugs or substance abuse, lived with an adult that was depressed or mentally ill, or attempted suicide, experienced violence or trauma (physical, psychological, or sexual abuse) or neglect, witnessed domestic violence. The total number of items children were reported to have experienced was summed into an adverse experience covariate (range: 0 to 4).

### Statistical analysis

Descriptive statistics were used to explore the data and calculate averages, spearman correlations, and percentiles of selected characteristics. The distributions of ΣBDE and ΣOPFRs were right skewed and subsequently natural log transformed. We then analyzed the data in two steps. First, we conducted multiple regression analysis to test for linear associations between levels of lnΣBDE or lnΣOPFR and subscales of social behaviors using Mplus version 6.0 with full information maximum likelihood estimation (FIML) [42]. These models were adjusted for children’s age, gender, flame retardant exposure, and family context. While the two flame retardant exposure variables were modestly correlated (ρ = 0.24) including both exposures in the model did not inflate standard errors by more than 5%. We then utilized generalized additive models (GAM) to explore non-linear associations between lnΣBDE or lnΣOPFR and subscales of social behaviors using R version 3.3 [43]. The upper limit on the degrees of freedom was set at k-1 to relax any assumptions about the shape of the exposure-response curve. Finally, all models were run with and without the flame retardant exposure variable to examine the proportion of variance explained by the chemical exposure.

## Results

Ninety-two children were recruited from 28 preschool classrooms in two geographic areas of Oregon (30% from site 1 and 70% from site 2). Descriptive statistics of the study population are presented in Table 1. Twenty-one percent of participants attended Head Start programs, a federal program in the U.S. that promotes school readiness for children living in poverty, and 79% attended community-based preschools. The racial/ethnic makeup of the sample was 79.3% White, 17.3% Non-white (5.4% African American, 7.6% Hispanic/Latino, 2.2% Asian, 1.1% Middle Eastern and 1.1% Native American), and 4.3% unknown race/ethnicity. Overall, this sample had similar proportions of low (34.8% completing High school/GED) and high (30.4% completing a doctorate degree (PhD, MD, JD)) levels of maternal education. Additionally, 3.3% of mothers completed an Associate’s degree, 22.8% completed a 4-year Baccalaureate degree, 1.1% completed a Master’s degree, and 7.6% declined to answer. The high rates of mothers with doctorate degrees is likely due to the fact that our recruitment sites occurred in small towns with universities. There were no significant differences in characteristics between those who did (*n* = 77) and did not (*n* = 15) return their wristbands to the lab for analysis.

**Table 1:** Descriptive statistics describing the study population

Variables	*N*	%Male	%Female		
Child gender	92	64%	36%		
		%No	%Yes		
Mother employed	86	34%	66%		
Father employed	60	12%	88%		
	*N*	*M*	*SD*	Min	Max
Child age in years	88	4.31	0.68	3.12	5.75
Family context					
Mother’s Education in years	86	16.30	3.67	10	34
Father’s Education in years	64	16.08	3.02	10	24
Household income^a^	86	5.22	2.84	1	8
Home learning environment^b^	88	0.01	1.00	−2.80	1.82
Adverse experiences	90	0.40	0.81	0	4
Flame retardants					
PBDE	72	3.54	1.15	0.42	5.96
OPFR	72	5.58	0.79	4.25	7.87
Teacher-rated social behavior					
Communication	89	1.94	0.50	0.29	3.00
Cooperation	89	1.96	0.61	0.67	3.00
Assertion	89	1.65	0.52	0.14	2.86
Responsibility	89	1.95	0.51	0.75	3.00
Empathy	89	1.93	0.58	0.50	3.00
Engagement	89	1.90	0.53	0.57	3.00
Self-control	89	1.73	0.61	0.14	3.00
Externalizing aggregate	89	0.76	0.48	0.00	2.06
Internalizing	89	0.61	0.47	0.00	1.86

^a^Household income is measured on a scale from 1 to 8

^b^Home learning environment is an aggregate of standardized items

Bivariate analysis revealed modest correlations between flame retardant exposure and some of the social behavior subscales (Table 2). We then used multiple regression analyses to further examine the association between flame retardant levels and social behavior subscales. We observed an association between flame retardant exposure and two of the positive social skills subscales, as well as the aggregate of externalizing behavior problems, controlling for child age, gender, family context, and adverse experiences (Table 3). More specifically, lnΣBDE levels were associated with less assertive behavior, as rated by preschool teachers (β = −0.31, *p* < 0.001), whereas lnΣOPFR levels were associated with both less responsibility (β = −0.25, *p* < 0.001) and more externalizing problems (β = 0.31, *p* < 0.05) after adjusting for gender, age, family context, and exposure to adverse health experiences. Interestingly, the magnitude of the effect between OPFR exposure and externalizing behavior was only slightly smaller than that of adverse experiences (β = 0.42, *p* < 0.001). Additionally, the magnitude of the effect between OPFR exposure and responsible behavior were similar, albeit in the opposite direction, of the association between the family context composite variable, which includes the home learning environment, and responsibility (β = 0.27, *p* < 0.001).

**Table 2:** Spearman correlation coefficients describing the bivariate associations between demographic information, flame retardant exposure, and social behaviors

Variable	Gender	Age	Family Context	Adverse Exper.	ln ΣPBDE	ln ΣOPFR	Communication	Cooperation	Assertion	Responsibility	Empathy	Engagement	Self-control	Externalizing	Internalizing
Gender	1														
Age	.06	1													
Family Context	− .09	.09	1												
Adverse Exper.	.03	.07	− .38**	1											
ln ΣPBDE	.08	.13	− .22^†^	.14	1										
ln ΣOPFR	.14	.02	− .15	.02	.24*	1									
Communication	.24*	.46**	.27*	− .19	− .12	− .10	1								
Cooperation	.24*	.27*	.24*	− .22*	.06	− .10	.77**	1							
Assertion	.15	.49**	.22*	− .13	− .33**	.09	.65**	.40**	1						
Responsibility	.24*	.33**	.32**	− .20^†^	.05	− .20^†^	.83**	.89**	.47**	1					
Empathy	.17	.37**	.16	− .19^†^	− .12	− .01	.69**	.52**	.57**	.56**	1				
Engagement	.13	.39**	.29**	− .23*	− .17	.06	.76**	.53**	.70**	.60**	.58**	1			
Self-control	.12	.39**	.39*	− .27*	− .20^†^	.05	.77**	.62**	.66**	.64**	.74**	.62**	1		
Externalizing	− .17	− .07	− .26*	.33**	.09	.26*	− .63**	− .78**	− .19^†^	− .74**	− .42**	− .41**	− .53**	1	
Internalizing	.20^†^	− .03	− .28**	.33**	.14	.09	− .35**	− .27**	− .12	− .26*	− .24*	− .32**	− .33**	.54**	1

^a^0 = male,1 = female

^†^ *p* < .10, *p < .05, **p < .01

**Table 3:** Multiple regression analyzes that examined the relationship between two classes of flame retardants and social behavior subscales (*n* = 69) adjusted for gender, age, family context, and child’s exposure to adverse experiences

	Assertion	Responsibility	Externalizing
	*B* (*SE*)®	*B* (*SE*)®	*B* (*SE*)®
Covariates			
Gender^a^	0.21 (0.10) 0.21*	0.44 (0.10) 0.43**	−0.29 (0.10) −0.30**
Age	0.32 (0.07) 0.44**	0.24 (0.07) 0.33**	−0.12 (0.10) −0.18
Family Context	0.13 (0.08) 0.18^†^	0.21 (0.08) 0.27**	−0.21 (0.11) −0.32^†^
Adverse Experiences	0.04 (0.07) 0.06	−0.04 (0.07) −0.05	0.31 (0.10) 0.42**
Flame Retardants			
Ln ΣPBDE	−0.13 (0.04) −0.31**	0.03 (0.04) 0.07	−0.05 (0.10) −0.04
Ln ΣOPFR	0.09 (0.06) 0.15	−0.16 (0.06) −0.25**	0.24 (0.10) 0.31*
R square	0.41	0.44	0.35
R square for model without Flame Retardant variables	0.28	0.29	0.19

^a^0 = male, 1 = female

*B* = Unstandardized Estimate. *SE* standard error. *®* = Standardized Estimate

^†^ *p* < .10. *p < .05. **p < .01

**Fig. 1:**
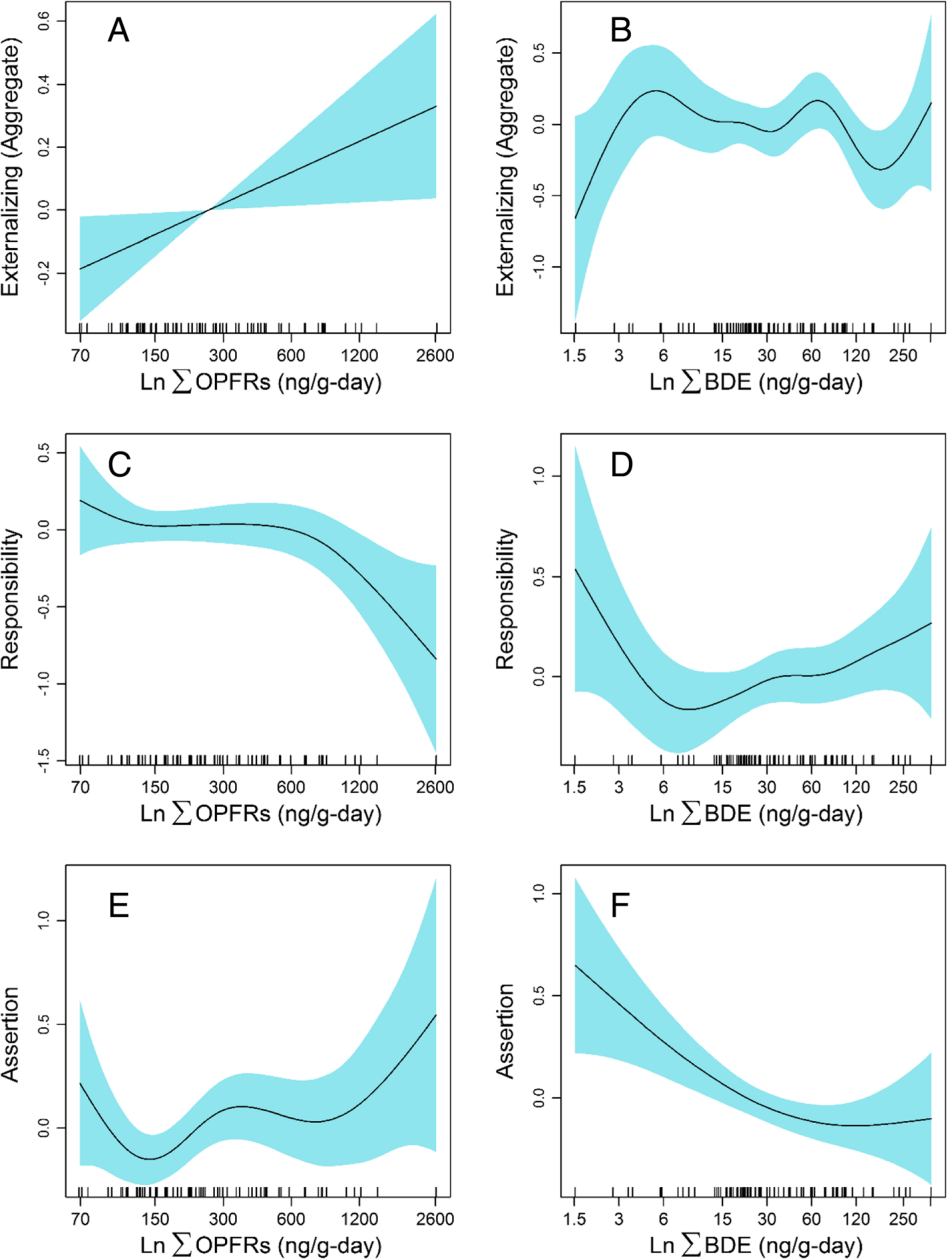
Exposure-response relationship between ln ΣOPFR ng/g-day and ln ΣBDE ng/g-day and externalizing behavior (**a**, **b**), responsibility (**c**, **d**), and assertion (**e**, **f**). All generalized additive models are adjusted for gender, age, family context, and child’s exposure to adverse experience(*n* = 69)

Generalized additive models were also used to examine the associations between flame retardant exposure and children’s social behaviors to examine the potential for non-linear exposure-responses (Fig. 1). This approach showed a positive, linear relationship between lnΣOPFR levels and children’s externalizing behaviors which explained 34.8% of the observed deviance controlling for child age, gender, family context, and adverse experiences (Fig. 1a, *p*-value = 0.03). We re-ran this model without lnΣOPFR to examine the proportion of the deviance explained by this exposure and noted that the adjusted R^2^ decreased to 29.4%. The exposure-response relationship between lnΣOPFR and responsible behavior was non-linear with the strongest effect among those children with the highest OPFR exposures (Fig. 1c, *p*-value = 0.07) after adjusting for covariates. The model which included lnΣOPFR explained 47.8% of the observed deviance, whereas the same model that did not include OPFRs decreased to 38.3%. No associations were observed between lnΣBDE levels and externalizing behavior (Fig. 1b, *p*-value = 0.30) or responsibility, as rated by preschool teachers (Fig. 1d, *p*-value = 0.24). However, a fairly linear association was observed between lnΣBDE levels and assertiveness, which appeared to plateau at the highest exposure levels after adjusting for age, gender, family context and adverse experience (Fig. 1f, *p*-value = 0.007). This model explained 46.6% of the observed deviance, whereas running the same model without lnΣBDE resulted in an observed deviance of 34.5%. Yet, no association was observed between lnΣOPFR levels and teachers’ ratings of children’s assertive behavior (Fig. 1e, *p*-value = 0.12).

## Discussion

Our findings indicated only modest associations between levels of exposure to PBDEs and OPFRs among preschool-aged children. This begs the question of whether one of these two families of flame retardants puts children at greater risk of developmental difficulties, such as with social behavior. The current study presents initial evidence that PBDEs and OPFRs relate to children’s development in different ways. In the current study OPFRs were predictive of more externalizing behavior problems, including aggression, defiance, hyperactivity, inattention, and bullying, as rated by children’s preschool teachers. The effect size for the association between exposure to flame retardants and these social skills were modest, but was similar in magnitude to the effect sizes for gender and family context, which are well-established predictors of externalizing problem behaviors [44, 45]. In the current study, only children’s early adverse experiences (e.g. abuse, neglect, parent mental illness/substance use) predicted externalizing behavior more strongly than OPFR levels. Children with higher OPFR levels were also rated by their preschool teachers as less responsible than children with lower OPFR levels, controlling for PBDE levels and covariates. The size of this association was modest; it was similar to the effect size for family context, but smaller than the effect sizes for child age and gender. Children with higher PBDE levels, on the other hand, were rated by their preschool teachers to be less assertive than were children with lower PBDE levels. It is notable that flame retardants were found to be linked with both more negative and less positive social behavior. Collectively this represents substantial risk for difficulty with academics, social relationships, and mental health [3, 4, 6, 7].

While our study employed a novel exposure assessment technology to measure personal exposure to flame retardants in early childhood [34] and was cross-sectional, our results are consistent with other larger prospective epidemiological studies that have observed an association between prenatal or early life BDE exposure and behavior in young children. For instance, Adgent et al. examined the association between exposure to PBDEs in breast milk at 3 months of age, an important route of exposure to these lipophilic compounds, and behaviors at 36 months of age using the Behavior Assessment System for Children, 2^nd^ edition [22]. This study reported that U.S. children with the highest exposure to PBDEs had higher levels of anxiety which is considered an internalizing problem. Another study examined the association between PBDEs in maternal blood collected during pregnancy and the child’s blood at age 9 and a suite of behavioral and cognitive outcomes measured at age 9 and 12 [29]. This study observed a significant association between higher PBDE levels and attention as measured by the Conners’ Continuous Performance Test II, processing speed as measured by the Weschler Intelligence Scales for Children, and executive function scores as measured by the Wisconsin Card Sorting Test and Behavior Rating Inventory of Executive Function. Another prospective study conducted in the US examined the association between PBDE levels in maternal blood collected during pregnancy and behaviors in children at age 5 to 8 years using the Behavior Rating Inventory of Executive Function [31]. This study observed that higher maternal serum PBDE levels were associated with poorer behavior regulation index scores. While the overall pattern of association between higher PBDE concentrations and poorer behavioral skills was observed in all of these studies including ours, the observed effect sizes for different PBDE congeners often differed in both magnitude and strength. These differences are likely due to differences in the study design and their ability to capture important biological and toxicological parameters such as the timing of exposures, composition of the chemical exposures, dose and duration of exposure, as well as the child’s age at the time of behavioral assessment. Another important difference is that the larger prospective cohorts were able to capture important critical windows of early neurodevelopment and utilized biomarkers of exposure which reduces the potential for misclassification.

Our study had several strengths, namely the use of the silicone passive sampler allowed us to measure children’s personal exposure to a mixture of flame retardants. This mixture included organophosphate-based flame retardants which have been rarely studied to date. Additionally, this sampler assessed exposure from wherever the children spent time and reflects both dermal and inhalation pathways. While the silicone passive sampler provides a measure of external exposures, validation studies show that the concentration of two OPFRs (TDCIPP and TCIPP) measured in the sampler were strongly correlated with their corresponding urinary metabolites (BDCIPP, ρ^2^_spearman_ = 0.59; and BCIPHIPP ρ^2^_spearman_ = 0.62) [46]. This suggests that the silicone wristband captures personal exposures over a short 5-day period. However, the silicone wristbands do not capture exposures from ingestion and would only reflect the exposures patterns present while it was being worn. This is a limitation to our study because our exposure assessment likely only captures children’s current flame retardant exposures which may differ from those experienced in utero or during other critical windows of development. Additionally, the exposures captured in the wristband would not reflect internal dose which can be measured in blood or urine. Thus, future studies should attempt to validate these findings using biological measurements of exposure. Our study also had other limitations including a cross-sectional design, a small sample size, and limited race/ethnic diversity, and high parent education levels. There is the potential that the limited diversity and/or high levels of parent education biased the associations between flame retardants and children’s developmental outcomes. For example, if a high level of parental education buffers children from deleterious effects of flame retardants on their development. Future research should examine potentially interactive effects between family characteristics such as parent education and flame retardants on children’s development. The use of teacher ratings of children’s social behaviors is also a limitation because ratings are not as objective as direct assessments, yet teacher ratings are also advantageous because they reliably capture variation in children’s behavior in classroom contexts that predicts their later success [47, 48]. Also, the seven subsets of positive behavior skills are strongly correlated, which is why we modeled them separately. However, this does introduce the potential for false positive results due to multiple comparisons. While this is a legitimate concern, the relationship between PBDEs and assertion, as well as OPFRs and responsibility remained statistically significant using a bonferroni correction (α = 0.05/7). Also, we obtained similar associations when modeling the data using GAMs.

## Conclusion

Children are exposed to different types of flame retardants from the built environment. These exposures have been associated with poorer attention and motor skills in children but less is known about how these compounds are related to children’s social skills. After controlling for social experiences and other factors, children with higher organophosphate flame retardant exposure were rated by their preschool teachers to show less responsible behavior and more externalizing behavior problems. Children with higher exposure to brominated flame retardants were rated by their preschool teachers as less assertive.

## Abbreviations

BDE: Brominated flame retardants
OPFR: Organophosphate flame retardants
PBDE: Polybrominated diphenyl ethers
SSIS-RS: Social skills improvement rating scale
